# Differentially expressed lncRNAs and mRNAs identified by microarray analysis in GBS patients *vs* healthy controls

**DOI:** 10.1038/srep21819

**Published:** 2016-02-22

**Authors:** Jing Xu, Chao Gao, Fang Zhang, Xiaofeng Ma, Xiaolin Peng, Rongxin Zhang, Dexin Kong, Alain R. Simard, Junwei Hao

**Affiliations:** 1 Department of Neurology, Tianjin Neurological Institute, Tianjin Medical University General Hospital, Tianjin 300052, China; 2 Laboratory of Immunology and Inflammation, Research Center of Basic Medical Science, Tianjin Medical University, Tianjin 300070, China; 3 Tianjin Key Laboratory on Technologies Enabling Development of Clinical Therapeutics and Diagnostics, School of Pharmacy, Tianjin Medical University, Tianjin 300070, China; 4 Département de Chimie et Biochimie, Université de Moncton, Moncton, New Brunswick, Canada

## Abstract

The aim of our present study was to determine whether message RNAs (mRNAs) and long noncoding RNAs (lncRNAs) are expressed differentially in patients with Guillain-Barré syndrome (GBS) compared with healthy controls. The mRNA and lncRNA profiles of GBS patients and healthy controls were generated by using microarray analysis. From microarray analysis, we listed 310 mRNAs and 114 lncRNAs with the mRMR software classed into two sample groups, GBS patients and healthy controls. KEGG mapping demonstrated that the top seven signal pathways may play important roles in GBS development. Several GO terms, such as cytosol, cellular macromolecular complex assembly, cell cycle, ligase activity, protein catabolic process, *etc*., were enriched in gene lists, suggesting a potential correlation with GBS development. Co-expression network analysis indicated that 113 lncRNAs and 303 mRNAs were included in the co-expression network. Our present study showed that these differentially expressed mRNAs and lncRNAs may play important roles in GBS development, which provides basic information for defining the mechanism(s) that promote GBS.

Guillain-Barré syndrome (GBS) is an acute inflammatory autoimmune disease affecting the peripheral nervous system. The characteristic features are progressive bilateral symmetric weakness and numbness in the legs and arms along with diminished or complete loss of deep tendon reflexes. In this presumed post-infectious, immune-mediated disease, cellular and humoral immune mechanisms probably play a vital developmental role. The production of autoantibodies or recruitment of inflammatory cells on the myelin sheath were thought to be responsible for the pathogenesis of GBS^1^. However, our present knowledge of the mechanism and epigenetic features of GBS remains insufficient.

Long noncoding RNAs (lncRNAs) are most commonly defined as transcripts longer than 200 nucleotides with little or no protein-coding capacity^2^,^3^. Since they cannot be completely dismissed as mere transcriptional “noise,” lncRNAs have attracted increasing attention based on the development of lncRNA microarrays, high-throughput sequencing, and bioinformatics^4^. Although without protein coding capability, accumulating evidence has suggested that lncRNAs participate in a wide variety of biological processes, including genomic imprinting, cell differentiation, chromosome modification, X-chromosome silencing, organogenesis, chromosome dosage-compensation, transcriptional activation, *etc*.^5^,^6^,^7^,^8^.

Currently, the role of lncRNA in autoimmune diseases has attracted considerable attention. Recent studies have reported that the activation, differentiation, and imbalanced expression of immune cells, including T cells, B cells, macrophages, and NK cells, may correlate directly with lncRNAs. Moreover, some specific lncRNAs also play a crucial role in autoimmune diseases such as systemic lupus erythematosus (SLE), rheumatoid arthritis (RA), psoriasis, and autoimmune thyroid disease (AITD)^9^.

Further, the molecular mechanisms underlying the contributions of lncRNAs to GBS are not clear. Therefore, in the present study, we applied microarray technology to examine lncRNA and message RNA (mRNA) expression profiles in blood samples from GBS patients and healthy controls. Additionally, results from gene ontology (GO) and Kyoto Encyclopedia of Genes and Genomes (KEGG) pathway analyses predicted that these abnormally expressed mRNAs and lncRNAs function in the development of GBS.

## Results

### lncRNA and mRNA expression profile in GBS patients

**Figure 1:**
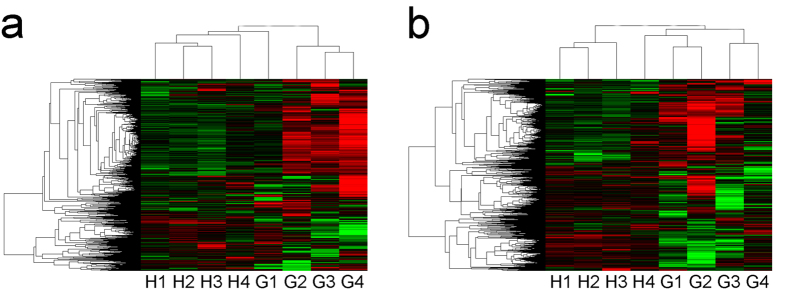
Hierarchical clustering of lncRNAs and mRNAs in GBS patients and healthy controls. G1-G4: GBS patients; H1-H4: healthy controls. The red and the green shades indicate the expression above and below the relative expression, respectively, across all samples. (**a**) lncRNA; (**b**) mRNA.

To investigate the expression levels of lncRNAs and mRNAs associated with GBS, lncRNA and mRNA microarray analyses were performed on the peripheral blood mononuclear cells (PBMCs) of 15 GBS patients and 15 healthy controls. Figure 1 was the hierarchical clustering that showed the differentially expressed lncRNAs (Fig. 1a) and mRNAs (Fig. 1b) between GBS patients and healthy controls. The red and the green shades indicate the expression above and below the relative expression, respectively, across all samples.

### Real-time quantitative PCR validation

**Figure 2:**
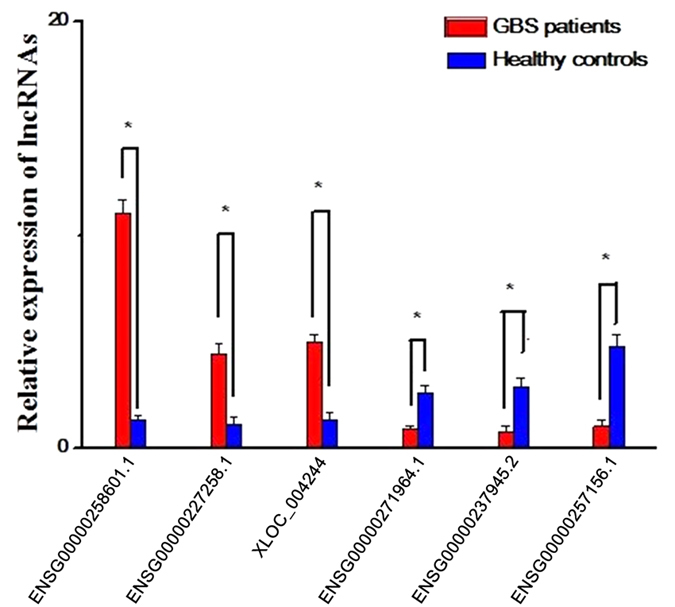
Validation of lncRNA microarray data by qRT-PCR. Three upregulated and three downregulated lncRNAs were validated by qRT-PCR of RNA extracted from PBMCs of 15 GBS patients and 15 healthy controls. The relative expression level of each lncRNA was normalized, and data displayed in histograms are expressed as means ± SD, *P < 0.05 comparing GBS patients with healthy controls.

To validate our results independently and determine the role of lncRNAs in GBS, we randomly selected 6 lncRNAs. As shown in Fig. 2, differences in the expression of 6 lncRNAs were detected in GBS patients compared with healthy controls. LncRNA ENSG00000258601.1 was the most elevated (8.1-fold higher expression), followed by lncRNA ENSG00000227258.1 (3.94-fold higher expression), and lncRNA XLOC_004244 (3.64-fold higher expression). LncRNA ENSG00000257156.1, lncRNA ENSG00000237945.2, and lncRNA ENSG00000271964.1 exhibited 4.58-, 3.72- and 2.96- fold lower expression, respectively. These results were consistent with the results obtained from the microarray chip analyses.

### Minimum Redundancy Maximal Relevance (mRMR) Result

After running the mRMR software, two outcomes were obtained. One was a MaxRel feature table ranking the 1246 mRNAs and 514 lncRNAs according to their relevance to the class of GBS patients or healthy controls (see File S1). The other, presented as the mRMR feature table, lists the top 310 mRNAs and 114 lncRNAs with the maximum relevance and minimum redundancy to the class of GBS patients or healthy controls (mRMR score equal 0 or 1, Table 1 and 2).

### GO and KEGG pathway analyses of differentially expressed mRNAs

**Figure 3:**
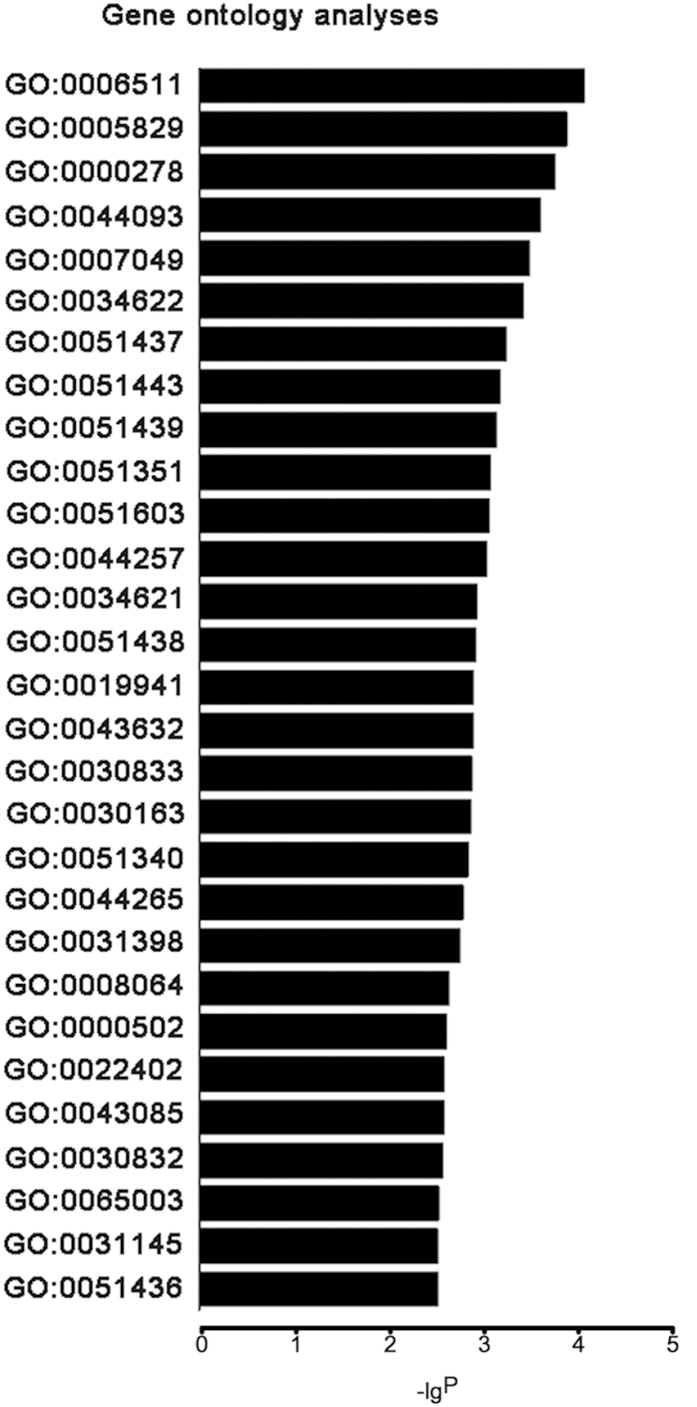
Top 29 gene ontology analysis. A total of 310 differentially expressed mRNAs were chosen based on the results of mRMR. The column graphs represent the enrichment of these mRNAs. The (−lg^P^) value was a positive correlation with GO. The (−lg^P^) values above 2.5 are presented. The top 29 GO are shown in detail in Table 3.

GO analysis was performed to investigate the over-representation of biological processes, cellular components, and specific molecular function associating protein-coding mRNAs, since no comprehensive annotation database is available for categorizing lncRNAs. A total of 310 filtered mRNAs (based on mRMR results) were included in GO analyses (see File S2). Figure 3 and Table 3 show the top 29 GO from the differentially expressed mRNAs (−lg^P^ > 2.5); these include cytosol, cellular macromolecular complex assembly, cell cycle, ligase activity, and protein catabolic process.

**Figure 4:**
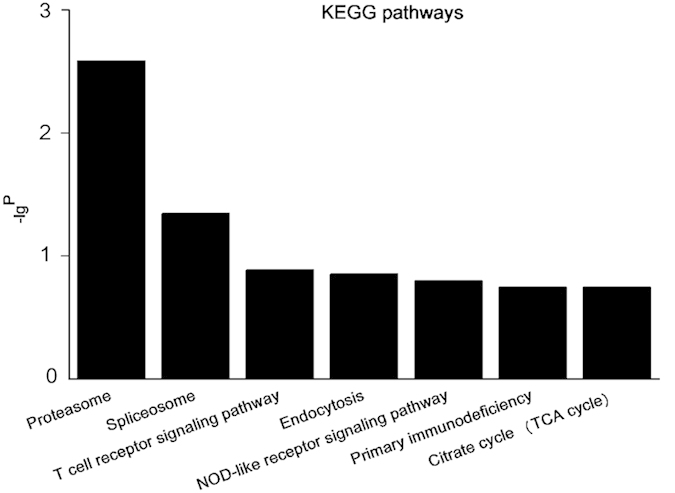
KEGG pathways. A total of 310 differentially expressed mRNAs were chosen based on the results of mRMR. The column graphs represent the enrichment of these mRNAs. The top seven significantly enriched KEGG pathways were calculated when plotted as the −lg^P^.

Furthermore, from the data in mRMR, top seven KEGG pathways were listed, as Fig. 4 depicts, including “Proteasome”, “Spliceosome”, “Citrate cycle (TCA cycle)”, “NOD-like receptor signaling pathway”, “Primary immunodeficiency”, “Endocytosis” and “T cell receptor signaling pathway.” Among them, “Proteasome” was the most significant, because it also appeared in the previous study^10^.

### lncRNA-mRNA co-expression network

**Figure 5:**
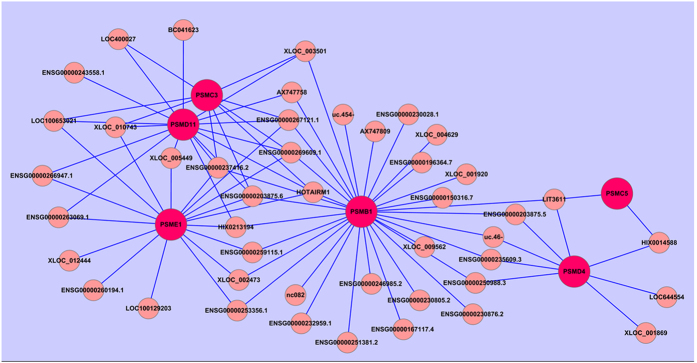
LncRNA-mRNA co-expression network in the “Proteasome” pathway. Here, 92 lncRNAs were interacting with 6 mRNAs in the meaningful “Proteasome” pathway.

Co-expression network analysis was performed between the 114 differentially expressed lncRNAs and the 310 differentially expressed mRNAs based on the mRMR results. In total, 113 lncRNAs and 303 mRNAs were included in the co-expression network. Moreover, our data showed that the co-expression network was composed of 5391 network nodes and 420 connections. The co-expression network indicated that one mRNA may correlate with 1–53 lncRNAs, and one lncRNA may correlate with 1 to 140 mRNAs (see File S3). Moreover, Fig. 5 reveals that 92 lncRNAs interacting with 6 mRNAs participated in the meaningful “Proteasome” pathway.

## Discussion

LncRNAs had long been considered as simply transcriptional noise^11^. However, recent studies showed that lncRNAs can regulate basal transcription, posttranscriptional processes, epigenetic modifications, DNA methylation, histone modification and even directly bind proteins, and regulate protein function^12^,^13^,^14^,^15^. Not until the last decade, however, has the discovery emerged that lncRNAs play an important role in diseases of the immune and nervous systems.

The first study implicating lncRNAs as regulators of the innate immune response showed that lincRNA-Cox2 is upregulated in mouse macrophages following exposure to lipopolysaccharide^16^. Subsequently, more lncRNAs were found to regulate the production of inflammatory mediators, such as LETHE, THRIL, NEAT1, PACER and IL-1β-RBT46^17^,^18^. A previous study focused on the involvement of lncRNA in modulating innate and adaptive immune responses, immune cell development, and differential expression of lncRNAs in autoimmune diseases^9^. In that context, although the pathogenesis of GBS has been extensively investigated, the exact molecular mechanism and epigenetic feature of this disease are still unclear. Therefore, establishing that lncRNA profiles are expressed differentially in GBS patients compared to their healthy counterparts is necessary and important.

In the present study, we investigated lncRNA and mRNA expression profiles in clinical samples from 15 GBS patients and 15 healthy controls using a microarray analysis. With mRMR software, we then ranked the mRNAs and lncRNAs according to their relevance to the class of GBS patients or healthy controls. The top 310 mRNAs and 114 lncRNAs were then identified according to their relevance to the class of GBS patients or healthy controls. These results indicated that these differentially expressed mRNAs and lncRNAs may be potential biomarkers for the diagnosis of GBS.

Based on the results of mRMR, GO and KEGG pathways, we proceeded to obtain detailed information on the biological functions and potential mechanisms of these mRNAs in GBS. GO analysis showed that these differentially expressed mRNAs based on mRMR results were enriched in top 29 GO (−lg^P^ > 2.5), including the cytosol, cellular macromolecular complex assembly, cell cycle, ligase activity, and protein catabolic process, etc (Fig. 3 and Table 3). As shown in Fig. 4, the top 310 mRNAs were associated with top seven major pathways, of which the “Proteasome” pathway was the most significant, as previously implicated in autoimmune diseases, especially GBS. The first report describing the role of proteasomes in autoimmune diseases noted that sera from patients with SLE contained specific autoantibodies against several polypeptide components of the proteasome^19^. Since then, patients with such autoimmune diseases as polymyositis-myositis and primary Sjogren’s syndrome also had autoantibodies against proteasomes^20^,^21^. Mengual *et al.* had shown that patients with multiple sclerosis (MS) presented with B and T cell autoreactivity against the proteasome in glial and neuronal cells^22^. Mayo *et al.* later wrote that both serum and cerebrospinal fluid (CSF) of MS patients had antibodies to almost all the polypeptide components of the proteasome. Additionally, their titres of these antibodies were 5-10-fold higher in the sera than in the CSF. Moreover, the incidence of anti-proteasome seroreactivity samples from MS patients was significantly higher than that in those from individuals with other inflammatory diseases, such as SLE, Sjogren’s syndrome, or sarcoidosis^23^. The previous study indicated that proteasome may be an antigenic target that evokes the cell-mediated immune response in MS patients and, possibly more generally, in several systemic inflammatory diseases.

GBS, as an acute inflammatory autoimmune disease affecting the peripheral nervous system, has attracted growing attention. Previous study showed that both the MB1 (X) and delta (Y) proteasome subunits were expressed in Schwann cells. Moreover, staining of the proteasome subunit delta (Y) was more abundant in peripheral nerves from GBS patients compared with those from inflammation-free controls^10^. Our present results from assessing the KEGG pathway in patients with GBS also indicated meaningful emphasis on the “Proteasome” pathway, an outcome that coincided with the previous studies^10^ and reinforced the veracity of our results.

The co-expression network analysis cited here was constructed based on the 114 differentially expressed lncRNAs and the 310 differentially expressed mRNAs, *i.e.,* in comparisons between GBS patients and healthy controls. Results showed that a total of 113 lncRNAs and 303 mRNAs were included in the co-expression network. This co-expression network, which was composed of 5391 network nodes and 420 connections, indicated that one lncRNA could target at most 140 mRNAs and one mRNA could correlate with at most 53 lncRNAs (see File S3). We also found that 92 lncRNAs interacted with 6 mRNAs involved in the meaningful “Proteasome” pathway (Fig. 5). This outcome suggests that the inter-regulation of lncRNAs and mRNAs is involved in the development of GBS and warrants further study.

In conclusion, the present study using microarray data provides newfound information regarding the potential role of mRNAs and lncRNAs in GBS patients. By using mRMR software, we also found top seven supposed KEGG pathways, especially a “Proteasome” pathway, and top 29 GO during GBS development. The co-expression network identified here also indicated the inter-regulation of lncRNAs and mRNAs in GBS patients. These findings may provide basic mechanistic information, possible biomarkers, and novel treatment strategies for patients afflicted with GBS.

## Experimental Procedures

### Patients and sample collection

For this study, we enrolled 15 GBS patients who fulfilled the standard diagnostic criteria for GBS in Tianjin Medical University General Hospital between 2014 and 2015^24^. When their blood was sampled, these patients were within the peak timing of manifesting GBS and before treatment with intravenous immune globulin (IVIG), plasma exchange or glucocorticoid. We also recruited 15 age- and gender-matched healthy controls for the comparative study. Informed consent was obtained at enrollment from all patients or legally acceptable surrogates. The study was carried out in accordance with the Declaration of Helsinki and with the Guide for Care and Use of Laboratory Animals as adopted and promulgated by the United National Institutes of Health. The present study was approved by the ethical review committees of Tianjin Medical University General Hospital. Peripheral blood anticoagulated by ethylene diamine tetraacetic acid (EDTA) was obtained from all GBS patients and healthy controls. Human PBMCs were isolated with Ficoll-Hypaque gradients.

### RNA extraction

For RNA purification, we used Trizol reagent (Invitrogen) according to the manufacturer’s instructions followed by application of PBMC to RNeasy spin columns (Qiagen, Venlo, Limburg, Netherlands). The RNA was quantified and the quality evaluated using a Nanodrop and Agilent 2100 Bioanalyzer (Agilent Technologies, Santa Clara, CA, USA), respectively. The individual RNA samples were stored at −80 °C until further use.

### Arraystar human lncRNA Microarray V3.0

The labeled cRNAs were hybridized onto the human LncRNA Expression Microarray V3.0 (Arraystar, Rockville, MD), which was designed for the global profiling of human lncRNAs and protein-coding transcripts. The third lncRNA microarray generated for each sample detected approximately 30586 lncRNAs and 26109 coding transcripts. Then, lncRNAs were carefully constructed using well-respected public transcriptome databases (Refseq, UCSC Known Genes, and Genecode), as well as landmark publications.

### Quantitative Real-time PCR validation

Real-time quantitative reverse transcription-polymerase chain reaction (qRT-PCR) is the gold standard for data verification. For the reverse transcriptase (RT) reaction, SYBR Green RT reagents (Bio-Rad, USA) were used. In brief, the RT reaction was performed for 60 min at 37 °C, followed by 60 min at 42 °C, using oligo (dT) and random hexamers. PCR amplifications were performed using SYBR Green Universal Master Mix. In brief, reactions were performed in duplicate containing 2× concentrated Universal Master Mix, 1 μL of template cDNA, and 100 nM of primers in a final volume of 12.5 μL, followed by analysis in a 96-well optical reaction plate (Bio-Rad). The lncRNA PCR results were quantified using the 2ΔΔct method against β-actin for normalization. The data represent the means of three experiments.

### mRMR method

The mRMR method was used to rank the importance of all features^25^,^26^,^27^. The mRMR method ranks these features based on not only their relevance to the target, but also the redundancy between features. A smaller index of a feature indicates that the latter index provides a better trade-off between maximum relevance to the target and minimum redundancy. The mutual information (MI) function, which estimates the extent to which one vector is related to another, quantifies both relevance and redundancy. The MI is defined as:


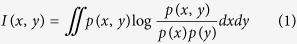


In equation (1), x and y are vectors, p(x, y) is their joint probabilistic density, and p(x) and p(y) are the marginal probabilistic densities. V supposedly denotes the entire feature set. Vs denotes the already-selected feature set containing m features, and Vt is used to denote the to-be-selected feature set containing n features. The relevance D between the target c and the feature f in Vt can be calculated by:


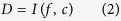


The redundancy R between all the features in Vs and the feature f in Vt can be calculated by:


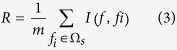


To determine the feature fj in Vt with maximum relevance and minimum redundancy, the mRMR function combines equation (2) and equation (3) and is defined as:


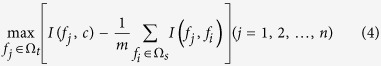


Then, the mRMR feature evaluation will continue N rounds when given a feature set with N (N = m+n) features. After evaluating the mRMR feature, a feature set S is obtained:


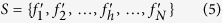


In this feature set S, the index h of each feature indicates at which round the feature is selected. The smaller the index h, the earlier the feature satisfies equation (4) and the better the feature is.

### GO and KEGG pathway analysis

GO was used to describe genes and gene product attributes, including cellular components, molecular functions, and biological processes. GO not only organizes genes into hierarchical categories but also uncovers the gene regulatory network on the basis of biologic processes and molecular functions^28^. KEGG mapping was used to predict the main pathways of the differentially expressed genes. DAVID method was used to select the main pathway with the significance threshold defined with *P* value and FDR^29^.

### Analysis of the lncRNA-mRNA co-expression network

Based on the correlation between the differentially expressed lncRNAs and mRNAs, the lncRNA-mRNA co-expression network was built. The network was constructed according to the normalized signal intensities of specific expression levels of mRNAs and lncRNAs. We used Pearson’s correlation coefficients, equal to or greater than 0.8, to identify the lncRNAs and coding genes. Then, the lncRNA-mRNA co-expression network was constructed by Cytoscape software (The Cytoscape Consortium, San Diego, CA, USA).

### Statistical analysis

All statistical data were analyzed with SPSS 17.0 software (SPSS Inc., Chicago, IL, USA). Differences in lncRNA expression between the GBS patients and healthy controls were analyzed using mRMR software. Statistical differences were considered significant at *P* < 0.05.

## Additional Information

**How to cite this article**: Xu, J. *et al.* Differentially expressed lncRNAs and mRNAs identified by microarray analysis in GBS patients *vs* healthy controls. *Sci. Rep.* **6**, 21819; doi: 10.1038/srep21819 (2016).

## Supplementary Material

Supplementary Dataset 1

Supplementary Dataset 2

Supplementary Dataset 3

## Figures and Tables

**Table 1:** Significant mRNAs based on mRMR result.

Order	mRNA	Order	mRNA	Order	mRNA
1	SLC35C1	35	CYTH4	69	FAM190B
2	LOC100507448	36	STK24	70	XRCC6
3	SLC35D1	37	SPTAN1	71	SEPW1
4	SLC35F2	38	STAT4	72	FAM160B1
5	SLC31A1	39	DCXR	73	FAM115C
6	ELFN1	40	DDOST	74	SEPHS1
7	ELF2	41	SNURF	75	SH3KBP1
8	ELMO1	42	B4GALT3	76	FER
9	ELL	43	SNRPD2	77	RYK
10	DTX3L	44	SNRPC	78	SACM1L
11	AUP1	45	SPOCK2	79	FBXO7
12	SLC9A6	46	DHX8	80	ZBTB2
13	ELMO2	47	SPATA21	81	RUFY2
14	ATP5O	48	SOX13	82	FGFRL1
15	ESCO1	49	FRYL	83	ASL
16	ESYT2	50	ZC3HAV1	84	SCNN1D
17	SLA2	51	ARNTL	85	FAM40A
18	EXOC3L1	52	ZBTB6	86	SDHD
19	SHOC2	53	RPS14	87	FBXO31
20	SHISA5	54	RRP1	88	GMEB1
21	EWSR1	55	FMN1	89	CCDC12
22	SKP1	56	RPL27	90	CCDC23
23	SLC25A39	57	RPN1	91	TSEN54
24	ATP6V1E1	58	ARPC4	92	TSPAN14
25	ATP8A1	59	RPS11	93	TSHZ1
26	EPB41	60	FNDC9	94	CASP5
27	DTNBP1	61	ZDHHC4	95	TUBA1B
28	SSBP4	62	ZDHHC20	96	CASP10
29	SSNA1	63	RNF113B	97	CARD10
30	DEFB1	64	RNASE3	98	CAPZA1
31	DDX19B	65	RIN3	99	C12orf57
32	DDX17	66	GDPD5	100	CD3E
33	SREBF1	67	CYP11A1	101	CD48
34	SRP14	68	FAM178B	102	CD244
Order	mRNA	Order	mRNA	Order	mRNA
103	TRIM56	139	CXCL5	175	LOC100130542
104	CCNK	140	SUSD1	176	NR3C1
105	CACNA2D4	141	XLOC_012444	177	ADAM12
106	C22orf46	142	CTAGE15P	178	ADD1
107	VCPIP1	143	TAP1	179	OBSCN
108	VAMP5	144	BIN1	180	OGDH
109	C21orf91	145	TACO1	181	LLPH
110	VDR	146	BROX	182	ADAR
111	VAMP2	147	XLOC_006443	183	PARP3
112	C3orf36	148	CEP350	184	PHC3
113	USP4	149	TOR1AIP1	185	PGRMC2
114	USP47	150	TP73	186	PFDN5
115	UTP18	151	CDK5RAP2	187	PI4K2B
116	VWCE	152	TOE1	188	KANSL2
117	VPRBP	153	CLASP1	189	KIF2A
118	C20orf201	154	CLIC5	190	KIF22
119	C19orf66	155	BRWD3	191	KIAA0947
120	USP39	156	TMEM104	192	KIAA1715
121	UBE2F	157	TM9SF2	193	KIAA1267
122	C9orf173	158	TLE4	194	ABCD3
123	UBP1	159	CHCHD3	195	MADD
124	UBE2E4P	160	CHMP4A	196	MPZL2
125	C5orf56	161	CIAPIN1	197	MAP2K7
126	C17orf85	162	CHRM4	198	MR1
127	C18orf25	163	ARHGAP30	199	LRP8
128	C6orf136	164	L2HGDH	200	LPGAT1
129	TCEB1	165	OR2A12	201	MAP3K4
130	COX17	166	P4HA2	202	LSM14A
131	TBC1D7	167	ZNF622	203	MIS12
132	TBCA	168	KLRB1	204	9-Sep
133	CNGB1	169	KRTAP10-3	205	MDH2
134	THRAP3	170	P4HB	206	MDM1
135	CORO7	171	PAPD7	207	METTL23
136	CLPS	172	NSMCE1	208	MED15
137	CWF19L1	173	LOC100127946	209	MESDC1
138	CTU2	174	LOC100130342	210	LOC731932
Order	mRNA	Order	mRNA	Order	mRNA
211	MAPKAP1	247	PSMD11	283	ILDR1
212	ABCA2	248	HCST	284	PPA1
213	MAPRE2	249	PVRL1	285	POLR2L
214	MAP3K7	250	HEATR7B1	286	PLXNA4
215	AAGAB	251	AP2A1	287	IDS
216	LOC100506191	252	Q9EPR2	288	ZNF350
217	LOC100506047	253	PSMD4	289	PLEKHA2
218	LOC100506906	254	PRKCB	290	IL10RA
219	NEK9	255	HLA-F	291	IKBKG
220	NGDN	256	HNRNPA1L2	292	AMPD2
221	NIPA2	257	HNRNPD	293	AMOTL1
222	ACTR3	258	APH1A	294	IL12RB1
223	NEUROG1	259	PSMB1	295	ANAPC13
224	ZNF728	260	HIST1H3C	296	ISG20L2
225	NCK1	261	PRR5	297	PPP1R2
226	LOC401480	262	PSMC3	298	PPP1R11
227	LOC644285	263	PSMC5	299	ITCH
228	LOC400128	264	PRPF6	300	ZNF24
229	MYBBP1A	265	HK1	301	PPP2R1A
230	MVP	266	ZNF207	302	HSP90AA2
231	MSH6	267	ARFGAP2	303	HSD17B10
232	MSRA	268	RB1CC1	304	ZNF26
233	LOC648044	269	GPR108	305	AMH
234	MYEOV2	270	GPN1	306	ISCU
235	NBAS	271	RBL2	307	PPIB
236	NAP1L4	272	GOLPH3	308	PMF1
237	ACOX2	273	GMPS	309	PPP2R5D
238	LOC200726	274	GNGT2	310	C1QL2
239	N4BP2	275	RASA3		
240	HERC6	276	GSTP1		
241	HERPUD2	277	GSPT2		
242	HIPK2	278	RAB11B		
243	HEG1	279	ZMYND11		
244	HINT2	280	RANGRF		
245	PSME1	281	RAB8A		
246	HBS1L	282	RAC1		

**Table 2:** Significant lncRNAs based on mRMR result.

Order	lncRNA	Order	lncRNA	Order	lncRNA
1	ENSG00000262967.1	35	XLOC_004629	69	FAM190B
2	ENSG00000263069.1	36	ENSG00000260194.1	70	XRCC6
3	DL492557	37	AX748067	71	SEPW1
4	ENSG00000234494.2	38	AX747809	72	FAM160B1
5	XLOC_001920	39	ENSG00000260550.2	73	FAM115C
6	ENSG00000234953.2	40	LOC644554	74	SEPHS1
7	XLOC_003501	41	HIX0014588	75	SH3KBP1
8	XLOC_003669	42	LOC729164	76	FER
9	XLOC_003365	43	HIX0032156	77	RYK
10	ENSG00000262721.1	44	LOC400027	78	SACM1L
11	CR936829	45	nc082	79	FBXO7
12	XLOC_001869	46	ENSG00000225407.3	80	ZBTB2
13	ENSG00000233044.1	47	LIT3556	81	RUFY2
14	ENSG00000232959.1	48	ENSG00000225886.1	82	FGFRL1
15	ENSG00000267121.1	49	LIT3584	83	ASL
16	ENSG00000266963.1	50	ENSG00000226266.2	84	SCNN1D
17	ENSG00000266947.1	51	LIT3611	85	FAM40A
18	ENSG00000266936.1	52	LOC100129203	86	SDHD
19	ENSG00000233138.1	53	ENSG00000272700.1	87	FBXO31
20	ENSG00000261609.1	54	HIX0213194	88	GMEB1
21	ENSG00000266677.1	55	ENSG00000227258.1	89	CCDC12
22	XLOC_000741	56	HOTAIRM1	90	CCDC23
23	ENSG00000167117.4	57	LOC100653021	91	TSEN54
24	ENSG00000150316.7	58	ENSG00000226849.1	92	TSPAN14
25	ENSG00000237416.2	59	LOC100652739	93	TSHZ1
26	XLOC_007231	60	ENSG00000269609.1	94	CASP5
27	ENSG00000259260.1	61	ENSG00000203875.6	95	TUBA1B
28	ENSG00000259115.1	62	ENSG00000203875.5	96	CASP10
29	ASO3749	63	ENSG00000269371.1	97	CARD10
30	AX747758	64	uc.263+	98	CAPZA1
31	ENSG00000235609.3	65	uc.46-	99	C12orf57
32	BC041623	66	uc.454-	100	CD3E
33	ENSG00000235586.1	67	ENSG00000267827.1	101	CD48
34	XLOC_005449	68	ENSG00000196364.7	102	CD244
Order	lncRNA				
103	ENSG00000242973.2				
104	ENSG00000244030.1				
105	AK311257				
106	XLOC_011769				
107	XLOC_011339				
108	ENSG00000249614.1				
109	ENSG00000249478.1				
110	ENSG00000243558.1				
111	AL833150				
112	ENSG00000255191.1				
113	AK289390				
114	XLOC_002473				

**Table 3:** Top 29 GO analyses.

Category	Term	Count	%	−lg^P^
GO:0006511	Ubiquitin-dependent protein catabolic process	14	4.73	4.044
GO:0005829	Cytosol	39	13.18	3.859
GO:0000278	Mitotic cell cycle	17	5.74	3.737
GO:0044093	Positive regulation of molecular function	22	7.43	3.589
GO:0007049	Cell cycle	26	8.78	3.469
GO:0034622	Cellular macromolecular complex assembly	15	5.07	3.409
GO:0051437	Positive regulation of ubiquitin-protein ligase activity during mitotic cell cycle	7	2.36	3.227
GO:0051443	Positive regulation of ubiquitin-protein ligase activity	7	2.36	3.159
GO:0051439	Regulation of ubiquitin-protein ligase activity during mitotic cell cycle	7	2.36	3.126
GO:0051351	Positive regulation of ligase activity	7	2.36	3.062
GO:0051603	Proteolysis involved in cellular protein catabolic process	21	7.09	3.046
GO:0044257	Cellular protein catabolic process	21	7.09	3.019
GO:0034621	Cellular macromolecular complex subunit organization	15	5.07	2.918
GO:0051438	Regulation of ubiquitin-protein ligase activity	7	2.36	2.911
GO:0019941	Modification-dependent protein catabolic process	20	6.76	2.882
GO:0043632	Modification-dependent macromolecule catabolic process	20	6.76	2.882
GO:0030833	Regulation of actin filament polymerization	6	2.02	2.862
GO:0030163	Protein catabolic process	21	7.09	2.857
GO:0051340	Regulation of ligase activity	7	2.36	2.826
GO:0044265	Cellular macromolecule catabolic process	23	7.77	2.772
GO:0031398	Positive regulation of protein ubiquitination	7	2.36	2.744
GO:0008064	Regulation of actin polymerization or depolymerization	6	2.03	2.625
GO:0000502	Proteasome complex	6	2.03	2.602
GO:0022402	Cell cycle process	19	6.42	2.576
GO:0043085	Positive regulation of catalytic activity	18	6.08	2.574
GO:0030832	Regulation of actin filament length	6	2.03	2.563
GO:0065003	Macromolecular complex assembly	21	7.09	2.519
GO:0031145	Anaphase-promoting complex-dependent proteasomal ubiquitin-dependent protein catabolic process	6	2.03	2.504
GO:0051436	Negative regulation of ubiquitin-protein ligase activity during mitotic cell cycle	6	2.03	2.504
